# Older Adults’ Contact Patterns with Family Members During COVID-19: Evidence from the NSHAP COVID Study

**DOI:** 10.1093/geroni/igaf122.239

**Published:** 2025-12-31

**Authors:** Xiaoyu Fu, Merril Silverstein, Catherine Garcia, Ying Xu, Tianqi Zhou

**Affiliations:** Syracuse University, Los Angeles, California, United States; Syracuse University, Syracuse, New York, United States; Syracuse University, Syracuse, New York, United States; Syracuse University, Syracuse, New York, United States; Syracuse University, Syracuse, New York, United States

## Abstract

The COVID-19 pandemic significantly disrupted older adults’ social connectedness, prompting many to adopt digital communication technologies to maintain family ties. This study examines distinct patterns of older adults’ family communication, including in-person visits, phone calls, and digital contact (email, social media, and video call), and retrospective assessments of changes in these interactions during the pandemic. Using data from the COVID sub-study of the National Social Life, Health & Aging Project (NSHAP), we applied latent class analysis to identify communication patterns among 2,048 adults aged 60 and over. Four distinct patterns emerged: (1) Moderate Contact with Decreased Frequency, characterized by moderate but declining engagement across all contact modes; (2) High Traditional Contact with Increased Frequency, marked by frequent in-person and phone contact with increases across all modes; (3) High Contact with Increased Digital Contact, defined by high engagement across all communication modes, particularly digital contact; and (4) Low Contact with Increased Frequency, indicating that although the frequency of contact has risen across all modes, overall contact levels remain low. These findings highlight the diverse ways older adults adapted their communication strategies during the pandemic, with some maintaining high levels of traditional contact, others expanding digital engagement, and some experiencing overall declines. As digital communication continues to play a crucial role in social connectedness, interventions should focus on enhancing digital literacy, increasing access to user-friendly technologies, and providing tailored support to help older adults sustain meaningful family interactions and mitigate social isolation.

